# Incidence of erectile dysfunction among middle-aged and aging sexual minority men living with or without HIV

**DOI:** 10.3389/fpubh.2024.1302024

**Published:** 2024-01-24

**Authors:** Aishat Mustapha, Brittanny M. Polanka, Mansi Maini, Deanna P. Ware, Xiuhong Li, Trevor A. Hart, Todd Brown, Frank Palella, Pamina M. Gorbach, Ken Ho, Michael Plankey

**Affiliations:** ^1^Department of Medicine, School of Medicine, Johns Hopkins University, Baltimore, MD, United States; ^2^Division of Epidemiology and Community Health, School of Public Health, University of Minnesota, Minneapolis, MN, United States; ^3^School of Medicine, Georgetown University, Washington, DC, United States; ^4^Department of Medicine, Georgetown University Medical Center, Washington, DC, United States; ^5^Department of Epidemiology, Bloomberg School of Public Health, Johns Hopkins University, Baltimore, MD, United States; ^6^Department of Psychology, Ryerson University, Toronto, ON, Canada; ^7^Department of Social & Behavioral Health Sciences, Dalla Lana School of Public Health, University of Toronto, Toronto, ON, Canada; ^8^Division of Infectious Diseases, Feinberg School of Medicine, Northwestern University, Chicago, IL, United States; ^9^Department of Epidemiology, Fielding School of Public Health, University of California, Los Angeles, Los Angeles, CA, United States; ^10^Department of Infectious Diseases and Microbiology, University of Pittsburgh, Pittsburgh, PA, United States

**Keywords:** sexual minority men, erectile dysfunction incidence, human immunodeficiency virus, multicenter AIDS cohort study, HIV

## Abstract

**Introduction:**

Erectile dysfunction (ED) has been established as a comorbidity among men living with HIV, but comparisons by HIV serostatus of ED incidence in a longitudinal follow-up cohort of men are lacking. We sought to evaluate the incidence of ED spanning a period of 12 years in a longitudinal cohort of sexual minority men (SMM) living with and without HIV.

**Methods:**

We analyzed ED incidence data for 625 participants in the longitudinal Multicenter AIDS Cohort Study from visits spanning October 2006 to April 2019.

**Results:**

SMM living with HIV were more likely to have incident ED compared with those living without HIV (rate ratio: 1.41; 95% CI: 1.14–1.75). Older age, current diabetes, cumulative cigarette use, and cumulative antidepressant use were associated with increased incidence of ED in the entire sample. Self-identifying as Hispanic, current diabetes, and cumulative antidepressant use were positively associated with ED incidence among SMM living with HIV. Cumulative cigarette use was positively associated with greater ED incidence only among SMM living without HIV.

**Discussion:**

In summary, age (full sample/ with HIV), current diabetes (full sample/with HIV), cumulative cigarette use (full sample/without HIV), and cumulative antidepressant use (full sample/with HIV) were associated with increased ED incidence. Skillful management of diabetes and careful titration of antidepressants, along with smoking cessation practices, are recommended to mitigate ED in this population.

## Introduction

The human immunodeficiency virus (HIV) was first described in 1981 and was quickly identified as a chronic infection with significant public health impacts. With the development of antiretroviral medications, the life expectancy for individuals with HIV has been extended. An increased lifespan for individuals with HIV has allowed for the identification of secondary effects of the illness, including erectile dysfunction (ED), and the inability to achieve and maintain an erection sufficient to complete satisfactory sexual activity. Several studies have characterized an increased prevalence among men living with HIV of 33–82%, as compared with 18% of men in the general population (1–5).

Demographic variables, comorbid conditions, and medication therapies have varying impacts on ED among men living with HIV. Specifically, older age and lower education levels have been associated with ED (6–8). Age is directly related to ED and hypogonadism, likely due to the premature decline of serum testosterone among men living with HIV (9). Low education levels are associated with ED, likely because of decreased access to medical care and related information to prevent sexual health decline (6, 8). Race, ethnicity, and household income have not been shown to have an association with ED among men living with HIV (6, 10, 11).

Several studies have established the prevalence of comorbid conditions among individuals with HIV and ED. Cardiovascular disease, viral hepatitis infection, and depression have been associated with a greater prevalence of ED among men living with HIV (10–15). Smoking is associated with ED among both the general population (1, 14) and men living with HIV (3). Some studies have shown an association of better erectile function among MLWH with higher CD4 counts (3, 7), although other studies have not (4, 6, 15). Cumulative years of antihypertensive medication use among men living with HIV has also been associated with increased prevalence of ED (16). Protease inhibitor use was significantly associated with sexual dysfunction among men living with HIV in one study (17) but not in others (14, 18). Increased duration of antiretroviral therapy has been shown to have a direct relationship with ED prevalence among men living with HIV including the associated risk of dyslipidemia (2, 14, 16); however, other studies did not find the same association due to small sample sizes, a lack of multivariable adjustment, or a limited focus on specific protease inhibitor medication (3, 4, 9).

To the best of our knowledge, most of the estimates of ED among men living with HIV have been based on prevalences; comparisons of ED incidence by HIV serostatus have been lacking. We undertook such an analysis in a longitudinally followed, multicity urban population of sexual minority men (SMM) followed up over 12 years.

## Materials and methods

The Multicenter AIDS Cohort Study (MACS) is a longitudinal study that examines the natural and treated history of HIV/AIDS among SMM living with or without HIV in Baltimore, Maryland/Washington, DC; Chicago, Illinois; Los Angeles, California; and Pittsburgh, Pennsylvania. A total of 7,352 SMM were enrolled over four time periods: 4,954 in 1984–1985, 668 in 1987–1991, 1,350 in 2001–2003, and 380 in 2011–2019. MACS participants attended semiannual visits that involved an audio-computer-assisted self-interview and a standardized clinic examination. Details on the inclusion and exclusion criteria for the MACS have been described elsewhere, but in brief, participants were HIV-negative or positive (HAART naïve or recent HAART initiation without a prior AIDS diagnosis) and reported sexual intercourse with men in the 5 years prior to enrollment. Participants living with HIV were excluded if the HIV infection was perinatally acquired or they had used injection drugs within 1 year of enrollment (19). This analysis included 625 SMM (90.9% gay, 3.5% bisexual, 2.1% heterosexual, and 3.5% other or prefer not to say) who were enrolled in the MACS through 2003 and did not report ED during study visits 46 (October 2006–March 2007) and 47 (April 2007–September 2007).

The presence of ED at visit 70 (October 2018–April 2019) was the primary outcome measure. ED was determined by summing the scores of a modified version of the six-item Erectile Functioning scale of the International Index of Erectile Function for gay and bisexual men. The scores ranged from 1 to 30, with scores of 17 or less indicating the presence of ED and scores >17 indicating the absence of ED (20).

Age at visit 70 was calculated using self-reported date of birth and was categorized into the following age groups: younger than 50, 50–54, 55–59, 60–64, 65–69, and 70 years and older. Race and ethnicity were self-reported and categorized as non-Hispanic white (reference group, hereafter referred to as white), non-Hispanic Black (hereafter referred to as Black), and Hispanic. Body mass index (BMI) at visit 70 was derived from weight and height and reported in kg/m^2^. Waist girth at visit 70 was obtained and reported in centimeters. Diabetes was defined as having a hemoglobin A_1c_ level of 6.5% or greater, a fasting glucose level of 126 mg/dL or greater, or a diagnosis of diabetes with the use of medication at visit 70 (21). Hypertension was defined as a systolic blood pressure of 140 mm Hg or greater, a diastolic blood pressure of 90 mm Hg or greater, or a diagnosis of hypertension with the use of medication at visit 70 (22). Current stimulant use (yes/no) was defined as any reported use of cocaine, crack, or methamphetamine at visit 70. Current cigarette smoking (yes/no) was defined as any reported use of cigarettes at visit 70. Current antihypertensive use (yes/no) was defined as the reported use of any antihypertensive medication at visit 70. Current antidepressant use (yes/no) was defined as the reported use of any antidepressant medication at visit 70. Current sexual-enhancing drug use, defined as any use of Cialis, Viagra, or Levitra (yes/no) was also reported. In addition to data on current use of substances and medications, cumulative cigarette packs per year, stimulant use, alcohol drinking, testosterone use, sexual-enhancing drug use, and highly active antiretroviral therapy use (for HIV-positive participants only) were calculated from visits 46 or 47 to visit 70. The level of plasma HIV RNA viral load was used to indicate HIV disease progression and was categorized as detectable (≥200 copies/ml) vs. undetectable (<200 copies/ml).

Descriptive statistics were generated for the outcome and independent variables using frequencies with percentages and medians with interquartile ranges as appropriate. Incidence rate was calculated as the percentage of ED assessed during visit 70 among men who did not have ED during visits 46 and 47. Poisson regression with robust error variance (23) was used to estimate incidence rate ratios (RRs) and 95% CIs of ED in multivariable models. Separate analyses were conducted for men with and without HIV, as well as the combined group, using the SAS version 9.2 GENMOD procedure (SAS Institute, Cary, NC, USA).

The covariates in the multivariable model for the SMM living without HIV and for the combined group included age, race and ethnicity, and current and cumulative use of alcohol, stimulants, cigarette smoking, antihypertensives, testosterone, sexual-enhancing drugs, and antidepressants. They were selected based on *a priori* published and expert knowledge (11). To compare the incidence rates by HIV serostatus, an additional model was performed combining both groups, including HIV serostatus and the covariates used in the model for SMM living without HIV. Given the possible association of BMI with ED, BMI was entered as a covariate in all analyses.

## Results

There were 625 participants (54.4% were living without HIV and 45.6% were living with HIV) included in the analysis. Descriptive statistics by HIV serostatus are reported in Table 1. Overall, 36% of participants reported ED at visit 70. The median age was 62 years (interquartile range, 57–68), 73% were white, 58% reported having hypertension, and 14% reported having diabetes. Current and cumulative use of alcohol, stimulants, cigarette smoking, antihypertensives, testosterone, sexual-enhancing drugs, and antidepressants is shown in Table 1.

**Table 1:** Characteristics of the study population by HIV status.

**Characteristics**	**No. (%)**		
	**HIV-positive**	**HIV-negative**	**Overall**
No. of men	285	340	625
Erectile dysfunction	119 (42%)	109 (32%)	228 (36%)
**Age (years)**			
Median (IQR)	60 (55–66)	64 (59–70)	62 (57–68)
<50	28 (10%)	17 (5%)	45 (7%)
50–54	39 (14%)	32 (9%)	71 (11%)
55–59	73 (26%)	59 (17%)	132 (21%)
60–64	63 (22%)	70 (21%)	133 (21%)
65–69	52 (18%)	74 (22%)	126 (20%)
≥70	30 (11%)	88 (26%)	118 (19%)
**Race and ethnicity**			
Black	73 (26%)	35 (10%)	108 (17%)
Hispanic	38 (13%)	21 (6%)	59 (9%)
white	174 (61%)	284 (84%)	458 (73%)
BMI, median (IQR)	26 (23–30)	27 (24–30)	26 (24–30)
Waist girth, median (IQR), cm	98 (90–109)	100 (91–109)	100 (90–109)
Current diabetes	44 (15%)	42 (12%)	86 (14%)
Current hypertension	173 (61%)	189 (56%)	362 (58%)
Current cigarette smoking	56 (20%)	30 (9%)	86 (14%)
Cumulative cigarette smoking pack-years, median (IQR)	0 (0–0.8)	0 (0–0)	0 (0–0)
Current stimulant use	39 (14%)	13 (4%)	52 (8%)
Cumulative stimulant use years, median (IQR)	0 (0–0.7)	0 (0–0)	0 (0–0)
Current antidepressant use	62 (22%)	52 (15%)	114 (18%)
Cumulative antidepressant use years, median (IQR)	0 (0–3.7)	0 (0–1.8)	0 (0–2.5)
**Current alcohol drinking^a^**			
None	70 (25%)	55 (16%)	125 (20%)
Low/moderate	1 (0–1)	1 (0–1)	1 (0–1)
Moderate/heavy	47 (16%)	49 (14%)	96 (15%)
Binge	11 (4%)	11 (3%)	22 (4%)
Cumulative alcohol drinking years, median (IQR)	12.5 (2.2–49.2)	21.4 (3.8–88.9)	16.3 (3–68.8)
Current testosterone use	43 (15%)	23 (7%)	66 (11%)
Cumulative testosterone use years, median (IQR)	0 (0–0.5)	0 (0–0)	0 (0–0)
Current sexual-enhancing drug use	66 (23%)	68 (20%)	134 (21%)
Cumulative sexual-enhancing drug use years, median (IQR)	0.5 (0–5)	0.5 (0–4)	0.5 (0–4.5)
Current HAART use	271 (95%)		
Cumulative HAART use years, median (IQR)	11.9 (10.5–12.3)		
HIV RNA ≥200 copies/ml	13 (5%)		
% CD4-positive cells, median (IQR)	35.5 (29.7–41)		

BMI, body mass index; HAART, highly active antiretroviral therapy; HIV, human immunodeficiency virus; IQR, interquartile range. ^a^Low/moderate drinking = 1–2 drinks/day or 3–4 drinks/day no more than once a month; moderate/heavy drinking = 3–4 drinks/day more than once a month or 5 or more drinks/day less than once a month; and binge drinking = 5 or more drinks/day at least once a month.

In the full sample, SMM living with HIV were 41% more likely to have ED at visit 70 compared to SMM living without HIV (RR: 1.41; 95% CI: 1.14–1.75; *p* = 0.002). A 5-year increase in age was associated with a 19% increase in the likelihood of ED (RR: 1.19; 95% CI: 1.09–1.29; *p* < 0.001). Current diabetes was associated with a 39% increase in the likelihood of ED (RR: 1.39; 95% CI: 1.10–1.75; *p* = 0.005). Every 10 years of cumulative cigarette smoking pack use was associated with a 33% increase in the likelihood of ED (RR: 1.33; 95% CI: 1.07–1.65; *p* = 0.009). Every 10 years of cumulative use of antidepressants was associated with a 51% increase in the likelihood of ED (RR: 1.51; 95% CI: 1.20–1.90; *p* < 0.001).

Among SMM living with HIV, age (RR: 1.21; 95% CI: 1.08–1.36; *p* = 0.001), Hispanic ethnicity (RR: 1.58; 95% CI: 1.04–2.41; *p* = 0.033), current diabetes (RR: 1.47; 95% CI: 1.09–1.97; *p* = 0.01), and cumulative antidepressant use (RR: 1.67; 95% CI: 1.23–2.27; *p* < 0.001) were associated with an increased likelihood of ED.

Among SMM living without HIV, age (RR: 1.15; 95% CI: 1.02–1.30; *p* = 0.024) and cumulative cigarette smoking (RR: 1.44; 95% CI: 1.04–2.00; *p* = 0.027) were associated with an increased likelihood of ED (Table 2).

**Table 2:** Incidence rate ratio over a 12-year period for erectile dysfunction using multivariable models.

**Factor**	**All men RR (95% CI)**	***p*-value**	**HIV-positive** **RR (95% CI)**	***p*-value**	**HIV-negative** **RR (95% CI)**	***p*-value**
HIV-positive vs. HIV-negative	**1.41 (1.14–1.75)**	**0.002**				
Age (5 years)	**1.19 (1.09–1.29)**	**<0.001**	**1.21 (1.08–1.36)**	**0.001**	**1.15 (1.02–1.30)**	**0.024**
Race and ethnicity						
Black vs. white	1.00 (0.75–1.34)	0.983	1.14 (0.8–1.62)	0.475	0.88 (0.48–1.61)	0.689
Hispanic vs. white	1.20 (0.84–1.70)	0.321	**1.57 (1.04–2.37)**	**0.032**	0.59 (0.23–1.52)	0.272
BMI (1 unit)	1.00 (0.95–1.05)	0.933	1.02 (0.96–1.08)	0.494	0.97 (0.89–1.05)	0.427
Waist girth (10 cm)	1.07 (0.90–1.27)	0.437	0.99 (0.8–1.23)	0.922	1.19 (0.89–1.60)	0.241
Current diabetes (yes vs. no)	**1.39 (1.10–1.75)**	**0.005**	**1.49 (1.11–1.98)**	**0.007**	1.24 (0.83–1.86)	0.284
Current hypertension (yes vs. no)	1.22 (0.97–1.53)	0.084	1.10 (0.80–1.5)	0.555	1.28 (0.90–1.81)	0.172
Cumulative cigarette smoking pack-years (10 years)	**1.33 (1.07–1.65)**	**0.009**	1.25 (0.87–1.81)	0.228	**1.44 (1.04–2.00)**	**0.027**
Cumulative stimulant use years (10 years)	1.03 (0.98–1.07)	0.257	1.03 (0.99–1.07)	0.145	0.99 (0.83–1.19)	0.942
Cumulative antidepressant use years (10 years)	**1.51 (1.20–1.90)**	**<0.001**	**1.62 (1.19–2.21)**	**0.002**	1.37 (0.95–1.99)	0.094
Cumulative alcohol drinking years (10 years)	1.00 (0.99–1.01)	0.961	0.99 (0.97–1.01)	0.514	1.00 (0.99–1.02)	0.70
Cumulative testosterone use years (10 years)	0.73 (0.42–1.27)	0.269	0.74 (0.39–1.41)	0.365	0.86 (0.29–2.54)	0.788
Cumulative sexual-enhancing drug use years (10 years)	0.79 (0.59–1.06)	0.123	0.73 (0.48–1.13)	0.158	0.87 (0.57–1.32)	0.503
Cumulative HAART use years (10 years)			1.36 (0.71–2.60)	0.35		
HIV RNA ≥200 vs. <200 copies/ml			0.88 (0.65–1.18)	0.39		
% CD4-positive cells (10%)			0.99 (0.84–1.17)	0.943		

BMI, body mass index; HAART, highly active antiretroviral therapy; HIV, human immunodeficiency virus; RR, rate ratio. Bold type highlights statistical significance at *p* < 0.05.

## Discussion

We found a significantly greater incidence of ED among SMM living with HIV compared with SMM living without HIV in a longitudinal follow-up cohort of urban SMM over a 12-year period. These findings corroborate previously published studies that demonstrated an increased prevalence of ED among men with HIV compared with the general population (1–5). Older age is known to be strongly associated with ED (9), with previous studies demonstrating that ED prevalence was <10% in men younger than 40 years and > 50% in men 40 years and older. In the current study, ED incidence increased with age and was most prevalent in men older than 60 years, regardless of HIV serostatus. In fact, we found an increase in ED of 19% with every 5 years of additional age.

Other factors associated with ED in our cohort differed by HIV serostatus. Among the SMM living with HIV, factors associated with ED incidence included race and ethnicity, diabetes, cumulative cigarette use, and cumulative antidepressant use. The association between race and ethnicity and ED is likely complex and multifactorial. Although only 9% of participants identified as Hispanic, we were able to detect a 57% greater ED incidence among Hispanic men with HIV relative to white men with HIV. Other studies have reported a greater prevalence of ED among African American populations (14). Variability across studies in findings regarding ED associations with race and ethnicity may be due to underlying structural and psychosocial factors that are more prevalent among certain racial and ethnic subgroups, requiring further study.

Diabetes has been implicated as a risk factor for ED among men with HIV with inconsistent findings. A proposed mechanism underlying the association of diabetes with ED is related to endothelial dysfunction and impaired nitric oxide synthesis (9). A 2020 systematic review of 14 studies examining factors associated with ED among men with HIV did not identify a significant association between diabetes and ED prevalence (10). This may have been due to differences in the baseline characteristics of our population. Diabetes, as it relates to ED, has also been studied in the context of metabolic syndrome because there is some overlap between risk factors associated with ED and generalized vascular or coronary artery disease (18). Our study indicated a 49% greater ED incidence among men with HIV and diabetes and a 39% greater incidence in the overall study population.

It is important to recognize the relationship between early vascular aging and HIV seropositivity. There is evidence that HIV is associated with metabolic abnormalities, endothelial dysfunction, increased artery stiffness, and stiffness of the carotid walls. Infection can result in premature cardiovascular disease because of viral injury, low-grade inflammation, and abnormal immune activation. Despite viral suppression by antiretroviral therapy, this dysfunction persists and leads to atherosclerosis, which is associated with erectile dysfunction (24).

Depression remains one of the most important factors that has been associated with ED in the general population. In one study, higher anxiety and depression scores were associated with ED (16). Depression has been shown to have direct and indirect effects on ED, including via antidepressant use, among men with and without HIV (11). The current study demonstrated a 62% increase in the incidence rate of ED among SMM living with HIV, comorbid depression, and cumulative concurrent antidepressant use. Our results are consistent with previously published findings addressing associations of the cumulative effects of antidepressant use on ED among participants in the MACS (17).

Notably, our findings did not indicate any statistically significant associations between ED and factors associated with HIV disease and treatment, including HIV viral suppression, CD4 count, and cumulative highly active antiretroviral therapy use. This finding is consistent with previous literature (10, 15).

Among men living with HIV in our cohort, cumulative cigarette smoking was associated with increased ED incidence. Current cigarette smoking appears to have a greater impact on the development of ED among men without HIV compared with the entire study population. Cumulative cigarette smoking was associated with increased ED incidence overall. Our findings are consistent with literature identifying smoking’s association with increased ED prevalence in the general population (1, 3, 16), likely mediated through the depletion of nitric oxide and vascular occlusion (25).

To date, findings from published data evaluating associations between stimulant and alcohol use with ED have been inconsistent. The role of stimulant use in ED is unclear; some studies have indicated a positive association with ED, while others have not indicated an association with ED among men living with HIV (10, 17). We found that stimulant use was not significantly associated with ED incidence in our cohort overall or among men living with HIV. A lack of significant results may be partially due to underreporting of stimulant use due to social stigma (11).

While this study is novel in its presentation of incidence RRs for ED in a large, multicity urban population, there were several limitations. First, the mean age of participants in our study was 62 years, and risk factors may not be generalizable to younger populations. Second, we did not stratify variables by severity of ED, nor were we able to confirm a diagnosis of ED beyond the use of the International Index of Erectile Function for gay and bisexual men. Third, we found an association between ED and Hispanic ethnicity among men living with HIV; this finding may have been influenced by our study sample, which included a low proportion of Hispanic individuals. Fourth, while our findings suggested the role of stimulant use in ED incidence among men living without HIV, we could not explore the underlying factors affecting stimulant use by HIV serostatus beyond depression. Varying reasons for stimulant use have been shown to have differing outcomes with respect to ED (11, 26). Finally, we did not have information on relationship status to differentiate between participants in exclusive relationships and those who were not.

To the best of our knowledge, the present study is the first to analyze the incidence of ED in a longitudinal follow-up, large urban population of SMM living with or without HIV. These findings are important given that ED can have a significantly negative impact on the affected individual’s quality of life, sometimes leading to depression and anxiety in settings requiring sexual performance and thus avoiding sexual relations (27–29). In many cases, ED and depression are intertwined and negatively reinforce each other. Individuals affected by ED may have decreased libido, and this may affect their relationship with sexual partners (30). The psychological consequences of ED may lead to avoidance of discussion and seeking treatment. For these reasons, screening for ED is important among SMM, independent of HIV. Given the risk factors associated with ED, such as diabetes and cigarette use, among others, screening among high-risk populations to enable counseling on lifestyle modification, referral to cognitive behavioral therapy in cases of comorbid mood disorders, careful titration of antidepressants by using the lowest effective dose, switching to alternate classes including selective norepinephrine reuptake inhibitors or norepinephrine/dopamine reuptake inhibitors, and reduction of the stigma associated with ED are important.

## Data availability statement

The datasets presented in this article are not readily available because data is available by request from the data analysis and coordination center at the Bloomberg School of Public Health at Johns Hopkins University. Requests to access the datasets should be directed to www.mwccs.org.

## Ethics statement

The studies involving humans were approved by Georgetown University, the UCLA School of Public Health, the Johns Hopkins University Bloomberg School of Public Health, the University of Pittsburgh Graduate School of Public Health, and the Northwestern University School of Medicine Investigational Review Boards. The studies were conducted in accordance with the local legislation and institutional requirements. The participants provided their written informed consent to participate in this study.

## Author contributions

AM: Writing – original draft, Writing – review & editing. BP: Writing – original draft, Writing – review & editing. MM: Conceptualization, Writing – review & editing. DW: Formal analysis, Writing – original draft, Writing – review & editing. XL: Data curation, Formal analysis, Software, Writing – review & editing. TH: Conceptualization, Investigation, Methodology, Writing – review & editing. TB: Conceptualization, Investigation, Writing – review & editing. FP: Conceptualization, Investigation, Writing – review & editing. PG: Conceptualization, Writing – review & editing. KH: Conceptualization, Writing – review & editing. MP: Conceptualization, Data curation, Formal analysis, Investigation, Methodology, Project administration, Supervision, Writing – review & editing.
